# Maternal Determinants of Birth Weight Variation Among Term Low Birth Weight Neonates in Taif, Saudi Arabia: A Cross-Sectional Study

**DOI:** 10.7759/cureus.115902

**Published:** 2026-09-07

**Authors:** Alhanouf Abdullah E. Almalki, Ghadi Nawaf Alotaibi

**Affiliations:** 1 Department of Pediatrics, King Faisal Medical Complex, Taif, SAU; 2 Department of Pediatrics, Taif Children Hospital, Taif, SAU

**Keywords:** birth weight, hemoglobin, low birth weight, maternal anemia, maternal determinants, maternal nutritional status, term neonates

## Abstract

Introduction: Low birth weight (LBW) remains an important maternal and neonatal health concern, including among infants delivered at term. This study assessed maternal factors associated with variation in birth weight among term LBW neonates in Taif, Saudi Arabia.

Methods: A cross-sectional study was conducted among 200 mothers who delivered term LBW neonates at Taif's Maternity Hospital, King Faisal Medical Complex. Maternal demographic, nutritional, hematological, medical, obstetric, and familial characteristics were assessed. Birth weight was analyzed as a continuous outcome using bivariate analyses and hierarchical multiple linear regression. Additional interaction analyses examined whether body mass index (BMI) and iron supplementation modified the association between maternal hemoglobin (Hb) and birth weight.

Results: The neonates had a mean birth weight of 2.20 ± 0.20 kg and a mean gestational age of 38.4 ± 1.22 weeks. Maternal anemia was present in 51.3% of participants, with a mean Hb concentration of 10.85 ± 1.66 g/dL. Maternal Hb was independently associated with birth weight (B = -0.028, 95% confidence interval (CI): -0.051 to -0.005; p = 0.020), while BMI showed a borderline association (B = 0.027, 95% CI: -0.002 to 0.056; p = 0.071). Consanguinity was independently associated with birth weight (B = 0.061, 95% CI: 0.004-0.118; p = 0.037). The final hierarchical model explained 14.2% of the variance in birth weight. The Hb × BMI and Hb × iron supplementation interaction terms did not significantly improve model fit.

Conclusions: Maternal Hb and consanguinity were independently associated with birth-weight variation among term LBW neonates, while BMI showed a weaker association. Further prospective studies are warranted to clarify these findings.

## Introduction

Anemia is characterized by a reduction in hemoglobin (Hb) concentration and a consequent decrease in the oxygen-carrying capacity of the blood, making it an important global public health concern [1,2]. Women of reproductive age are among the population groups most affected, with the World Health Organization (WHO) estimating that approximately one-third have anemia [3]. Anemia has diverse etiologies, including nutritional deficiencies of iron, vitamin A, vitamin B12, folate, and riboflavin, as well as inherited disorders such as sickle-cell disease and thalassemia [4,5]. During pregnancy, anemia is one of the most common maternal comorbidities worldwide [6]. In Saudi Arabia, the prevalence of maternal anemia was estimated at 27.3% in 2019 [7]. The WHO defines anemia during pregnancy as an Hb concentration below 11 g/dL [8]. Maternal Hb status has important implications for pregnancy outcomes; both low and very high maternal Hb concentrations have been associated with adverse outcomes in some studies, while severe anemia, particularly Hb levels below 6 g/dL, has been associated with poor pregnancy outcomes [1,9], including stillbirth, fetal anemia, and low or very low birth weight (LBW) [10].

LBW is an important indicator of maternal and neonatal health and is associated with substantial morbidity, mortality, and longer term health consequences [11]. The WHO defines LBW as a birth weight of less than 2,500 g [12]. Globally, approximately 15%-20% of births are estimated to be LBW, corresponding to more than 20 million births annually, with the majority occurring in low- and middle-income countries [13]. LBW is associated with an increased risk of neonatal complications and mortality [14], as well as adverse developmental outcomes, including cognitive impairment, motor developmental delay, cerebral palsy, and behavioral and psychological difficulties [15,16]. LBW may also contribute to adverse health outcomes extending from infancy and childhood into adulthood [17,18], while its broader consequences impose substantial social and economic burdens on healthcare systems and societies [19,20]. Birth weight is also an important predictor of neonatal survival and subsequent health [21]. Therefore, identifying maternal factors associated with fetal growth and birth weight remains an important priority for maternal and neonatal health.

Birth weight is influenced by multiple maternal, obstetric, nutritional, and familial factors. Maternal Hb has received particular attention because impaired maternal oxygen-carrying capacity may affect placental and fetal oxygenation. However, the relationship between maternal Hb and birth weight is not entirely consistent, with previous studies reporting varying associations across different maternal Hb concentrations and pregnancy stages [22,23]. Maternal nutritional status may similarly influence fetal growth, while factors such as maternal age, parity, iron supplementation, medical conditions, antenatal care, previous pregnancy outcomes, multiple pregnancy, placental and umbilical cord abnormalities, infection, and premature rupture of membranes (PROMs) may also contribute to birth-weight variation. Considering these factors simultaneously may provide a more comprehensive understanding of maternal determinants than examining individual characteristics in isolation.

In Saudi Arabia, evidence on maternal determinants of LBW remains limited and inconsistent, particularly regarding the relationship between consanguinity and neonatal anthropometry. Moreover, previous studies have often examined LBW as a binary outcome or included preterm and term births together, limiting the ability to distinguish factors associated with fetal growth among term LBW neonates. Evidence specifically addressing birth-weight variation among term LBW neonates in Taif is particularly limited.

Therefore, the primary objective of this study was to investigate maternal factors independently associated with birth-weight variation among term LBW neonates delivered at Maternity Hospital-King Faisal Medical Complex (KFMC), Taif, Saudi Arabia. Secondary objectives were to assess the associations of maternal Hb, body mass index (BMI), iron supplementation, demographic, obstetric, medical, and familial characteristics with birth weight, and to examine the incremental contribution of maternal characteristic groups and potential interactions between Hb, BMI, and iron supplementation.

## Materials and methods

Study design and setting

A cross-sectional observational study was conducted to investigate maternal factors associated with variation in birth weight among term LBW neonates delivered at Maternity Hospital-KFMC, Taif, Saudi Arabia. The study was conducted over an eight-month period.

Study population and eligibility criteria

The study population comprised women who delivered term neonates between 37 and 42 completed weeks of gestation with a birth weight below 2.5 kg during the study period. LBW was defined as a birth weight of <2,500 g (<2.5 kg). All eligible mother-neonate records meeting these criteria during the study period were included. Both singleton and multiple pregnancies were retained because multiple pregnancy was one of the maternal and obstetric characteristics examined in the study. Records without a valid birth-weight measurement were excluded from analyses involving the primary outcome. For other variables, analyses were conducted using the available observations, with the number of observations reported where applicable.

Sample

The final analytical dataset comprised 200 mother-neonate records meeting the study eligibility criteria. The study population represented all eligible records available during the specified study period.

Data collection and study variables

Maternal and neonatal information was extracted from medical records using a standardized data collection form. Maternal variables included maternal age, educational/social status, nutritional status/BMI category, Hb concentration, iron supplementation, medical illness, consanguinity, gravidity, parity, history of abortion/stillbirth, family history of LBW, and antenatal care. Pregnancy and obstetric variables included multiple pregnancy, placenta and umbilical cord abnormalities, oligohydramnios, and maternal infection or PROMs. Neonatal variables included sex, birth weight, and gestational age at delivery.

Birth weight was treated as the primary continuous outcome variable and was recorded in kilograms. Maternal Hb was analyzed as a continuous variable in grams per deciliter. Maternal anemia was additionally classified as Hb <11 g/dL. Maternal nutritional status was analyzed according to the BMI categories recorded in the medical records. Iron supplementation, consanguinity, antenatal care, multiple pregnancy, medical illness, family history of LBW, placenta/cord abnormalities, and maternal infection/PROM were analyzed as categorical variables. Gravidity and parity were considered measures of obstetric history and were categorized for descriptive analyses and appropriately coded for regression analysis.

Data management and quality control

Data were entered into Microsoft Excel (Microsoft Corporation, Redmond, WA) and subsequently reviewed for completeness, consistency, coding errors, duplicate records, and implausible values before being imported into IBM Statistical Package for the Social Sciences (SPSS) Statistics, version 26 (IBM Corp., Armonk, NY). Inconsistencies in terminology, capitalization, and categorical responses were standardized during data cleaning. Continuous variables were checked for plausible ranges and consistency with the original medical records. Missing values were coded as missing rather than replaced with arbitrary values. Corrections to previously incomplete gestational-age records were made where the medical records confirmed the gestational age.

Statistical analysis

Statistical analyses were performed using IBM SPSS Statistics, version 26. Categorical variables were summarized using frequencies and percentages. Continuous variables were presented as mean ± standard deviation when approximately normally distributed and as median with interquartile range when distributions were nonnormal. Distributional characteristics were assessed using graphical methods and the Kolmogorov-Smirnov test.

Because all participants delivered term LBW neonates, birth weight was analyzed as a continuous outcome rather than as a binary LBW outcome. Differences in birth weight between two-category maternal variables were assessed using the Mann-Whitney U test, while the Kruskal-Wallis test was used for comparisons involving more than two categories.

A multivariable linear regression model was used to evaluate the independent associations between maternal and obstetric characteristics and continuous neonatal birth weight. The regression model incorporated clinically relevant demographic, maternal, nutritional, obstetric, and medical characteristics. Associations were presented as unstandardized regression coefficients (B) with 95% confidence intervals (CIs) and p values. For continuous predictors, coefficients represented the expected change in birth weight associated with a one-unit increase in the predictor, whereas categorical predictors were interpreted relative to their specified reference categories.

A hierarchical multiple linear regression analysis was performed to assess the incremental explanatory contribution of different groups of maternal characteristics. Demographic characteristics were entered in the first block, followed by maternal biological and nutritional characteristics, including Hb, BMI, and iron supplementation, and subsequently obstetric and medical characteristics. The change in explained variance (ΔR²) between successive models was examined to determine whether each additional block significantly improved the explanatory capacity of the model.

An interaction analysis was conducted to examine whether the association between maternal Hb and birth weight differed according to maternal BMI or iron supplementation. Specifically, Hb × BMI and Hb × iron supplementation interaction terms were entered into the regression model. The statistical significance of the interaction terms and the corresponding change in explained variance were assessed.

The assumptions of linear regression were evaluated before interpretation of the final models. These included linearity, independence of observations, homoscedasticity, normality of residuals, multicollinearity, and influential observations. Multicollinearity was assessed using tolerance and variance inflation factor values, while residual plots and regression diagnostics were used to assess model assumptions and identify potentially influential observations.

All statistical tests were two-sided, and a p value of <0.05 was considered statistically significant. Effect estimates were reported with 95% CIs.

Ethical considerations

The study was approved by the relevant research ethics committee in the Kingdom of Saudi Arabia under KACST Committee Registration Number H-02-T-123 and Approval Number 2025-E-66. The study was conducted in accordance with applicable ethical requirements and regulations governing research involving human participants in Saudi Arabia. As the study involved the review of existing medical records, the requirement for individual informed consent was waived. All data were deidentified before analysis, and confidentiality and privacy were maintained throughout data collection, data management, analysis, and reporting.

## Results

A total of 200 mothers with a mean age of 31.1 ± 6.6 years, with 22.7% aged 18-25 years, 26.3% aged 26-30 years, 25.3% aged 31-35 years, and 25.8% aged 36-46 years, were included. The majority of women reported a moderate educational or social status (59%), with only a small proportion being either uneducated (2.5%) or reporting a good status (2.5%). About half of the women (50.5%) did not receive antenatal care.

More than half of the mothers (51.3%) were anemic, while 48.7% had normal Hb levels. Among the anemic group, moderate anemia was the most common (31.7%), followed by mild anemia (19.1%), whereas severe anemia was rare (0.5%). The overall mean Hb level was 10.85 ± 1.66 g/dL, with a range of 6.3-15.0 g/dL. About half (44.5%) of mothers had a normal BMI, while 20% were overweight, and 16% were obese.

Regarding comorbidities, gestational diabetes mellitus (GDM) was present in 9% of women, hypertension in 7%, preeclampsia in 5.5%, and hypothyroidism in 3.5%. A family history of LBW was found in 31%, and consanguinity was observed in 41%. Over half of the women (52%) reported taking iron supplements during pregnancy.

The obstetric history revealed that the majority (43.5%) were multigravida and 44% were multiparous, with a mean gravidity of 3.34 ± 2.42 and a mean parity of 1.86 ± 1.84. Notably, 27.8% of the women reported experiencing pregnancy losses, whereas 13.9% experienced recurrent losses. Abnormalities of the placenta or umbilical cord were observed in 8.5% of cases, and oligohydramnios in 0.5%. Additionally, maternal infections or PROMs were noted in 15% of the participants. All details are represented in Table 1.

**Table 1:** Maternal characteristics among women who delivered term babies at Taif's Maternity Hospital-KFMC ^*^Other: Rh-negative, SLE, DVT, and HBV-positive BMI: body mass index; GDM: gestational diabetes mellitus; HTN: hypertension; PET: preeclampsia; PROM: premature rupture of membranes; LBW: low birth weight; SLE: systemic lupus erythematosus; DVT: deep vein thrombosis; HBV: hepatitis B virus; SD: standard deviation; IQR: interquartile range; KFMC: King Faisal Medical Complex

Factor	Category	Value
Age (years) (n = 198)	18-25, n (%)	45 (22.7)
	26-30, n (%)	52 (26.3)
	31-35, n (%)	50 (25.3)
	36-46, n (%)	51 (25.8)
	Mean ± SD	31.1 ± 6.6
	Median (IQR)	31 (10)
	Range	18-46
Education level/social status, n (%)	Good	5 (2.5)
	Moderate	118 (59)
	Low	72 (36)
	Uneducated	5 (2.5)
BMI (kg/m^2^), n (%)	Underweight (<18.5)	39 (19.5)
	Normal (18.5-24.9)	89 (44.5)
	Overweight (25-30)	40 (20)
	Obese (>30)	32 (16)
Hemoglobin (g/dL) (n = 199)	Normal (≥11), n (%)	97 (48.7)
	Mild (10-10.9), n (%)	38 (19.1)
	Moderate (7-9.9), n (%)	63 (31.7)
	Severe (<7), n (%)	1 (0.5)
	Mean ± SD	10.85 ± 1.66
	Median (IQR)	10.9 (2.3)
	Range	6.3-15
Comorbidities, n (%)	Yes	45 (22.5)
	No	155 (77.5)
Specify, comorbidities, n (%)	GDM	18 (9)
	HTN	14 (7)
	PET	11 (5.5)
	Hypothyroidism	7 (3.5)
	Bronchial asthma	2 (1)
	Other^*^	9 (4.5)
Family history of LBW, n (%)	Yes	62 (31)
	No	138 (69)
Iron supplement, n (%)	Yes	104 (52)
	No	96 (48)
Consanguinity, n (%)	Yes	82 (41)
	No	118 (59)
Gravity	Primigravida (1), n (%)	59 (29.5)
	Multigravida (2-4), n (%)	87 (43.5)
	Grand multigravida (≥5), n (%)	54 (27)
	Mean ± SD	3.34 ± 2.42
	Median (IQR)	3 (4)
	Range	1-13
Parity	Nullipara (0), n (%)	63 (31.5)
	Primipara (1), n (%)	34 (17)
	Multipara (2-4), n (%)	88 (44)
	Grand multipara (≥5), n (%)	15 (7.5)
	Mean ± SD	1.86 ± 1.84
	Median (IQR)	2 (3)
	Range	0-10
Abortion/stillbirth (n = 180)	None, n (%)	130 (72.2)
	Once, n (%)	25 (13.9)
	Recurrent (≥2), n (%)	25 (13.9)
	Mean ± SD	0.54 ± 1.13
	Median (IQR)	0 (1)
	Range	0-7
Multiple pregnancy, n (%)	Yes	18 (9)
	No	182 (91)
Placenta and cord abnormalities, n (%)	Yes	17 (8.5)
	No	183 (91.5)
Oligohydramnios, n (%)	Yes	1 (0.5)
	No	199 (99.5)
Maternal infection/PROM, n (%)	Yes	30 (15)
	No	170 (85)
Antenatal care, n (%)	Yes	99 (49.5)
	No	101 (50.5)

All neonates were full-term and LBW, with a mean LBW of 2.2 ± 0.2 kg. Among term neonates, 58.5% were female and 41.5% male neonates (Table 2).

**Table 2:** Neonatal characteristics among women who delivered term babies at Taif's Maternity Hospital-KFMC SD: standard deviation; IQR: interquartile range; KFMC: King Faisal Medical Complex

Factor	Category	Values
Gender, n (%)	Female	117 (58.5)
	Male	83 (41.5)
Birth weight (kg)	Mean ± SD	2.2 ± 0.2
	Median (IQR)	2.24 (0.28)
	Range	1.48-2.44
Gestational age (weeks)	Mean ± SD	38.4 ± 1.22
	Median (IQR)	38 (2)
	Range	37-42

The relationship between maternal factors and neonatal birth weight is presented in Table 3. Maternal Hb level approached but did not reach statistical significance (p = 0.077) in its impact on LBW, with anemic mothers having a slightly lower median birth weight of 2.23 (0.30) kg compared with 2.25 (0.30) kg in nonanemic mothers. Similarly, maternal BMI showed a comparable borderline pattern (p = 0.077), where obese mothers had higher birth weights than those with lower BMIs. Consanguinity was the only factor with a statistically significant association with LBW (p = 0.002), where infants of consanguineous parents had a higher median birth weight of 2.30 (0.25) than 2.20 (0.23) kg in those without consanguinity.

**Table 3:** The impact of maternal and obstetric factors on neonatal birth weight among term deliveries ^*^Mann-Whitney test ^⸸^Kruskal-Wallis test LBW: low birth weight; IQR: interquartile range; BMI: body mass index; PROM: premature rupture of membranes

Factor	Category	Birth weight (kg), median (IQR)	p value
Maternal anemia (g/dL)	Yes (<11)	2.23 (0.3)	0.077^*^
	No (≥11)	2.25 (0.3)	
Age (years)	18-25	2.20 (0.31)	0.446^⸸^
	26-30	2.25 (0.30)	
	31-35	2.25 (0.30)	
	36-46	2.20 (0.22)	
Education level/social status	Good	2.20 (0.16)	0.674^⸸^
	Moderate	2.25 (0.29)	
	Low	2.22 (0.24)	
	Uneducated	2.30 (0.23)	
BMI (kg/m^2^)	Underweight (<18.5) (coded as 1)	2.20 (0.20)	0.077^⸸^
	Normal (18.5-24.9) (coded as 2)	2.20 (0.27)	
	Overweight (25-30) (coded as 3)	2.20 (0.45)	
	Obese (>30) (coded as 4)	2.30 (0.20)	
Comorbidities	Yes	2.20 (0.35)	0.430^*^
	No	2.25 (0.28)	
Family history of LBW	Yes	2.23 (0.25)	0.975^*^
	No	2.25 (0.30)	
Iron supplement	Yes	2.25 (0.28)	0.519^*^
	No	2.20 (0.25)	
Consanguinity	Yes	2.30 (0.25)	0.002^*^
	No	2.20 (0.23)	
Gravity	Primigravida	2.25 (0.28)	0.736^⸸^
	Multigravida	2.23 (0.27)	
	Grand multigravida	2.24 (0.30)	
Parity	Nullipara	2.20 (0.28)	0.952^⸸^
	Primipara	2.30 (0.40)	
	Multipara	2.25 (0.29)	
	Grand multipara	2.20 (0.25)	
Abortion/stillbirth	None	2.23 (0.26)	0.786^⸸^
	Once	2.25 (0.35)	
	Recurrent	2.20 (0.30)	
Multiple pregnancy	Yes	2.25 (0.26)	0.561^*^
	No	2.23 (0.28)	
Placenta and cord abnormalities	Yes	2.30 (0.23)	0.108^*^
	No	2.20 (0.25)	
Maternal infection/PROM	Yes	2.28 (0.26)	0.271^*^
	No	2.20 (0.28)	
Antenatal care	Yes	2.25 (0.28)	0.815^*^
	No	2.22 (0.30)	

As shown in Table 4, hierarchical multiple linear regression analysis was performed to examine the incremental contribution of maternal demographic, biological, and obstetric characteristics in predicting neonatal birth weight. In Model 1, maternal demographic variables (maternal age, gestational age, and educational/social status) explained 1.5% of the variance in birth weight (R² = 0.015). Following the addition of maternal biological characteristics (Hb concentration, BMI, and iron supplementation) in Model 2, the explained variance increased to 7.2% (ΔR² = 0.057), representing a statistically significant improvement over Model 1 (p = 0.009). Incorporating obstetric and medical variables in Model 3 further increased the explained variance to 13.1% (R² = 0.131). Finally, the inclusion of the interaction terms (Hb × BMI and Hb × iron supplementation) in Model 4 resulted in a final explained variance of 14.2% (R² = 0.142; adjusted R² = 0.051). However, the interaction block did not significantly improve the model (ΔR² = 0.011, p = 0.330), indicating that maternal BMI and iron supplementation did not significantly modify the relationship between maternal Hb concentration and neonatal birth weight.

**Table 4:** Hierarchical multiple regression model summary Hb: hemoglobin; BMI: body mass index

Model	Variables added	R²	Adjusted R²	ΔR²	p value for change
Model 1	Maternal age, gestational age, education	0.015	0.000	0.015	0.529
Model 2	+ Hemoglobin, BMI, iron supplementation	0.072	0.043	0.057	0.009
Model 3	+ Obstetric and medical variables	0.131	0.050	0.059	0.082
Model 4	+ Hb × BMI, Hb × Iron	0.142	0.051	0.011	0.330

The final hierarchical regression model identified maternal Hb concentration and consanguinity as independent predictors of neonatal birth weight (Table 5). Lower maternal Hb concentration remained significantly associated with lower neonatal birth weight (B = -0.028, p = 0.020), whereas consanguinity demonstrated a positive association with birth weight (B = 0.061, p = 0.037) after adjustment for demographic, biological, obstetric, and medical characteristics. Maternal BMI showed a borderline positive association with birth weight (B = 0.027, p = 0.071). Maternal age, gestational age, parity, iron supplementation, GDM, hypertension, preeclampsia, hypothyroidism, family history of LBW, multiple pregnancy, placenta or cord abnormalities, maternal infection/PROM, and antenatal care were not independently associated with neonatal birth weight (all p > 0.05). Furthermore, the Hb × BMI and Hb × iron supplementation interaction terms were not statistically significant, indicating no evidence that maternal nutritional status or iron supplementation modified the relationship between maternal Hb concentration and birth weight.

**Table 5:** Final hierarchical multiple regression model (model 4) CI: confidence interval; BMI: body mass index; GDM: gestational diabetes mellitus; HTN: hypertension; PET: preeclampsia; LBW: low birth weight; PROM: premature rupture of membrane

Predictor	B	95% CI	p value
Maternal hemoglobin (g/dL)	-0.028	-0.051 to -0.005	0.020
Maternal age (years)	0.001	-0.004 to 0.006	0.709
Gestational age (weeks)	0.009	-0.011 to 0.029	0.372
BMI	0.027	-0.002 to 0.056	0.071
Consanguinity	0.061	0.004 to 0.118	0.037
Iron supplementation	0.018	-0.041 to 0.078	0.544
GDM	-0.021	-0.122 to 0.080	0.684
HTN	-0.083	-0.205 to 0.038	0.179
PET	-0.108	-0.222 to 0.006	0.063
Hypothyroidism	-0.012	-0.155 to 0.131	0.869
Family history of LBW	0.019	-0.042 to 0.080	0.538
Parity	-0.011	-0.028 to 0.007	0.239
Multiple pregnancy	0.034	-0.066 to 0.135	0.501
Placenta/cord abnormalities	0.081	-0.025 to 0.188	0.135
Maternal infection/PROM	0.038	-0.046 to 0.122	0.374
Antenatal care	0.023	-0.037 to 0.082	0.454

## Discussion

LBW remains an important marker of adverse intrauterine conditions and is associated with increased neonatal morbidity and mortality as well as longer term health consequences. Although prematurity is a major pathway to LBW, fetal growth restriction among infants delivered at term represents a distinct clinical phenomenon. The present study therefore provides a focused assessment of maternal factors associated with variation in birth weight among term LBW neonates in Taif, Saudi Arabia. This population is particularly relevant because maternal anemia, nutritional characteristics, pregnancy complications, patterns of antenatal care, and familial factors may coexist and contribute to fetal growth through interconnected pathways.

Maternal Hb is one of the most consistently investigated maternal determinants of fetal growth. The biological basis for an association is plausible because inadequate maternal Hb may reduce oxygen-carrying capacity and potentially compromise placental and fetal oxygenation. Maternal anemia may also represent broader nutritional or inflammatory disturbances that affect fetal development. A systematic review and meta-analysis of 71 studies found that maternal anemia was associated with LBW (adjusted OR: 1.23, 95% CI: 1.06-1.43), supporting anemia as an important maternal risk factor for adverse fetal growth [24]. More recent global evidence has strengthened this association. An updated systematic review and meta-analysis of 148 studies found that low maternal Hb was associated with LBW, small-for-gestational-age birth, preterm birth, stillbirth, and neonatal mortality [25]. Importantly, the review also demonstrated that the magnitude and direction of associations varied according to the timing of Hb assessment and the threshold used to define low or high Hb, highlighting the complexity of the relationship between maternal Hb and fetal growth.

The Saudi context provides additional support for the relevance of maternal hematological status. A large study from Abha involving 7,067 antenatal records identified maternal anemia among the factors associated with LBW and also reported an association between inadequate antenatal care and LBW [26]. More recent Saudi research on extremely LBW has likewise emphasized maternal health, antenatal care, and nutritional status as important areas for prevention [27]. Taken together, these findings support continued attention to maternal hematological status within antenatal services in Saudi Arabia.

At the same time, the relationship between Hb and fetal growth should not be interpreted as a simple linear pathway. Maternal Hb normally changes across pregnancy because of physiological plasma-volume expansion, and both low and unusually high concentrations have been associated with adverse pregnancy outcomes. The global systematic review by Young et al. identified associations between both low and high Hb concentrations and adverse outcomes and demonstrated that these associations varied by gestational timing and Hb thresholds [25]. This is particularly important when interpreting the present study because the analysis was restricted to term LBW neonates. Consequently, the study addresses variation in birth weight within a clinically selected LBW population, rather than estimating the probability of LBW in the general population. This distinction should be maintained when interpreting the maternal Hb association and prevents the findings from being incorrectly interpreted as evidence that higher Hb increases the population risk of LBW.

Maternal nutritional status is another important component of fetal growth. BMI is a readily available indicator but represents only one dimension of maternal nutritional and metabolic health. Extensive evidence indicates that maternal underweight is associated with increased risk of LBW, while higher maternal BMI is more commonly associated with larger fetal size and other metabolic or obstetric complications. A systematic review and meta-analysis of more than one million pregnancies found that maternal underweight was associated with increased risk of LBW, with the association observed in both developed and developing countries [28]. Similarly, a large meta-analysis from China found that maternal underweight increased the risk of LBW, whereas higher maternal BMI was more strongly associated with fetal overgrowth and other adverse neonatal outcomes [29].

The present findings should therefore be considered within the broader, nonlinear relationship between maternal nutritional status and fetal growth. BMI alone cannot distinguish between differences in maternal height, gestational weight gain, dietary quality, micronutrient availability, or metabolic health. This is especially relevant in settings experiencing nutritional transition, where maternal obesity and nutritional deficiencies can coexist. Future research in Saudi Arabia would benefit from incorporating prepregnancy BMI, gestational weight gain, dietary intake, maternal height, and biochemical indicators of nutritional status rather than relying on BMI as the principal measure of maternal nutrition.

Consanguinity represents a particularly important consideration in the Saudi setting. The prevalence and social importance of consanguineous marriage make it relevant to the investigation of reproductive and neonatal outcomes. Nevertheless, existing evidence regarding its relationship with fetal growth is inconsistent. A large Saudi study involving 4,497 newborns found no significant differences in birth weight, height, head circumference, chest circumference, or mid-arm circumference according to the degree of parental consanguinity and concluded that consanguinity did not have a significant effect on fetal growth [29]. More importantly, a study conducted specifically in Taif among 1,468 newborns similarly found no significant differences in birth weight, height, or head circumference across first-cousin, second-cousin, and unrelated couples [30]. That study included assessment of maternal and gestational factors as well as socioeconomic indicators, further suggesting that the relationship between consanguinity and neonatal anthropometry is not straightforward.

The divergence between these previous observations and the association identified in the present study is therefore noteworthy. It should not, however, be interpreted as evidence that consanguinity has a protective effect on fetal growth. Differences in study populations, outcome definitions, degrees of parental relatedness, socioeconomic characteristics, reproductive histories, and other unmeasured factors may contribute to the observed heterogeneity. Most importantly, the present study specifically examined term LBW neonates, whereas the previous Taif investigation assessed newborn anthropometry across a broader birth-weight distribution. Restriction of the outcome to term LBW infants may therefore reveal patterns that are not apparent in an unselected newborn population. The finding warrants replication in larger, multicenter Saudi cohorts in which the degree and type of consanguinity can be examined alongside genetic, socioeconomic, and maternal nutritional characteristics.

Antenatal care is an important potential pathway for modifying maternal risk factors because it provides opportunities for the detection and treatment of anemia, nutritional deficiencies, hypertensive disorders, diabetes, infection, and other pregnancy complications. Previous Saudi evidence has reported an association between inadequate antenatal care and LBW, while research in other settings has similarly emphasized the importance of timely and adequate antenatal services [26]. However, antenatal care should not be conceptualized simply as attendance vs. nonattendance. Its potential effect depends on when care begins, the number and continuity of visits, screening procedures, nutritional interventions, management of pregnancy complications, and the quality of care received. A binary measure may therefore underestimate the contribution of antenatal care to fetal growth. This consideration is particularly important when comparing the present findings with studies that used more detailed measures of antenatal-care adequacy.

The same principle applies to other maternal and obstetric characteristics. Conditions such as gestational diabetes, hypertension, preeclampsia, thyroid disease, infection, placental abnormalities, and PROMs can influence fetal growth through different mechanisms, but their effects depend on disease severity, timing, treatment, and coexistence with other maternal factors. The absence of a consistent independent association for individual clinical characteristics should therefore not be interpreted as evidence that these conditions are clinically unimportant. Rather, their contribution may be heterogeneous and may require larger samples, more detailed clinical characterization, and longitudinal assessment to distinguish independent effects from correlated maternal risk factors.

The hierarchical modeling approach provides an important conceptual contribution because maternal determinants of fetal growth do not operate in isolation. Demographic characteristics may influence nutritional status, access to healthcare, reproductive history, and maternal health, while biological characteristics such as Hb and BMI may themselves reflect several underlying processes. Sequential modeling of these domains allows assessment of whether maternal biological characteristics contribute information beyond demographic characteristics and whether obstetric and medical factors provide additional explanatory value. This approach is particularly appropriate for a multifactorial outcome such as birth weight and provides a more informative framework than examining each maternal characteristic independently.

The interaction analysis similarly addresses the possibility that maternal factors may modify one another's effects. The implications of maternal Hb could plausibly differ according to nutritional status, while the relationship between Hb and fetal growth could potentially depend on iron supplementation. However, interaction effects are generally more difficult to detect than main effects and require adequate sample sizes within the relevant exposure strata. In addition, a binary measure of iron supplementation does not capture dosage, duration, adherence, timing, or the clinical indication for treatment. Consequently, future studies should investigate these relationships using longitudinal measures of Hb and iron status, along with more detailed information on supplementation.

The clinical implications of these findings should be considered within the broader maternal-health framework. The consistent international evidence linking maternal anemia and low Hb with adverse birth outcomes supports continued emphasis on early identification and appropriate management of maternal anemia during pregnancy. However, anemia management should not be separated from comprehensive nutritional and antenatal care. Hb concentration alone does not establish iron deficiency, and effective prevention requires consideration of the underlying cause of anemia, dietary intake, adherence to supplementation, and other maternal health conditions. In Saudi Arabia, integrating these elements into antenatal care may provide a more effective strategy than focusing on any single maternal characteristic.

Several strengths should be considered when interpreting the study. The restriction to term LBW neonates provides a focused examination of determinants of birth-weight variation without prematurity being the primary explanation for LBW. The study also incorporated a broad range of demographic, nutritional, obstetric, medical, and familial characteristics. The use of hierarchical regression permitted assessment of the incremental contribution of groups of maternal factors, while interaction analysis allowed examination of potential effect modification. Furthermore, the study addresses a relatively underinvestigated population: term LBW neonates in Taif rather than simply reproducing the extensive literature on LBW as a binary outcome.

The study also has important limitations. Its single-center, record-based design may limit generalizability to other regions and healthcare settings in Saudi Arabia and may introduce potential selection or information bias. The sample size of 200 participants may also have limited statistical power, particularly for uncommon clinical conditions and interaction analyses. In addition, some variables had incomplete observations, which may have introduced bias despite analyses being conducted using available data. Maternal Hb was assessed without complementary biomarkers such as serum ferritin, limiting the ability to distinguish iron-deficiency anemia from other causes of low Hb. Detailed information on prepregnancy nutritional status, gestational weight gain, dietary intake, maternal height, placental function, and other potentially important determinants was also limited. Finally, the observational cross-sectional design precludes causal inference; therefore, the findings should be interpreted as associations within the study population rather than causal or general population-level effects.

## Conclusions

This study examined maternal determinants of birth weight among term LBW neonates in Taif, Saudi Arabia. The findings indicate that maternal Hb is an important independent maternal factor associated with variation in birth weight, while maternal BMI showed a weaker association and consanguinity demonstrated a context-specific association that warrants further investigation. Overall, the findings support a multifactorial approach to addressing term LBW, emphasizing comprehensive antenatal assessment, maternal hematological and nutritional health, and consideration of population-specific factors. Further multicenter prospective studies incorporating detailed measures of iron status, maternal nutrition, gestational weight gain, and consanguinity are recommended to strengthen the evidence and guide targeted prevention strategies in Saudi Arabia.
